# Advances in the Understanding of the Lifecycle of Photosystem II

**DOI:** 10.3390/microorganisms10050836

**Published:** 2022-04-19

**Authors:** Virginia M. Johnson, Himadri B. Pakrasi

**Affiliations:** Department of Biology, Washington University, St. Louis, MO 63130, USA; virginia.johnson@wustl.edu

**Keywords:** Photosystem II, oxygenic photosynthesis, photosynthetic reaction center, cyanobacteria, cryo-electron microscopy

## Abstract

Photosystem II is a light-driven water-plastoquinone oxidoreductase present in cyanobacteria, algae and plants. It produces molecular oxygen and protons to drive ATP synthesis, fueling life on Earth. As a multi-subunit membrane-protein-pigment complex, Photosystem II undergoes a dynamic cycle of synthesis, damage, and repair known as the Photosystem II lifecycle, to maintain a high level of photosynthetic activity at the cellular level. Cyanobacteria, oxygenic photosynthetic bacteria, are frequently used as model organisms to study oxygenic photosynthetic processes due to their ease of growth and genetic manipulation. The cyanobacterial PSII structure and function have been well-characterized, but its lifecycle is under active investigation. In this review, advances in studying the lifecycle of Photosystem II in cyanobacteria will be discussed, with a particular emphasis on new structural findings enabled by cryo-electron microscopy. These structural findings complement a rich and growing body of biochemical and molecular biology research into Photosystem II assembly and repair.

## 1. Introduction: Photosystem II and Cyanobacteria

Photosystem II (PSII) is a multi-subunit membrane protein complex located in the thylakoid membrane of plants, cyanobacteria, and algae. It is the enzyme responsible for splitting water, providing the oxygen that fuels life on Earth as we recognize it. PSII performs this demanding photochemical reaction through sequential oxidation of a catalytic Mn_4_CaO_5_ cluster, which accumulates four oxidizing equivalents, cycling through five so-called S_n_-states of oxidation [1] before oxidizing water in a concerted manner into molecular oxygen, producing four protons and four electrons in the process.

Cyanobacteria, oxygenic photosynthetic prokaryotes, have frequently been used as model organisms to study oxygenic photosynthetic processes due to their relatively fast growth and ease of genetic manipulation as compared with plants. For study of Photosystem II, three organisms have been widely utilized: *Synechocystis* sp. PCC 6803 (S6803), *Thermosynechococcus elongatus*, and *Thermosynechococcus vulcanus.* The mesophilic S6803 has been utilized for physiological studies and studies that require genetic manipulation of photosynthetic proteins, as it is a facultative heterotroph. The ability to grow S6803 on glucose allows for mutations and deletions to be made in genes necessary for photoautotrophic growth. PSII from either *T. elongatus* or *T. vulcanus*, thermophilic cyanobacterial strains, has been utilized for all high-resolution X-ray diffraction structures of PSII. Proteins from thermophilic organisms are often used for structural studies, as these proteins are more stable than their mesophilic homologs. Currently, due to these structural studies, our ‘static’ picture of active PSII is quite detailed. Atomic-resolution X-ray diffraction structures are available from *T. elongatus* and *T. vulcanus* [2,3,4] and X-ray free-electron laser (XFEL) technology and sample preparations have advanced to allow the structural determination of each of the Kok’s S-states of the Mn_4_CaO_5_ cluster up to S_3_ [5]. Recently, cryo-electron microscopy has enabled elucidation of the structure of active PSII from S6803 as well [6].

The biogenesis and repair (PSII lifecycle) of this membrane-protein-pigment complex are under active investigation. The assembly of PSII, a 20+ subunit membrane-protein-pigment complex, is an intricate process, involving cellular localization of protein subunits, pigment insertion, subunit assembly and processing, and cofactor assembly. Furthermore, under normal growth conditions, PSII is routinely damaged by reactive oxygen species that form within the protein and in its immediate vicinity [7,8]. To maintain a high level of photosynthetic activity, PSII must be repaired when damage occurs: damaged protein subunits must be replaced while the undamaged ones remain protected, and the biogenesis and repair processes must be coordinated within the cell.

In this review, we will discuss what is known about PSII assembly and repair in cyanobacteria, with an emphasis on new findings since the topic was last reviewed [9,10,11,12,13,14,15,16,17]. Recently, the field has been greatly advanced by high-resolution cryo-electron microcopy (cryo-EM). Cryo-EM has enabled several PSII assembly intermediate structures to be solved, as well as structures from the mesophilic S6803. These advances, in addition to other progress in the field, warrant a review and discussion.

## 2. PSII Structure

**Figure 1:**
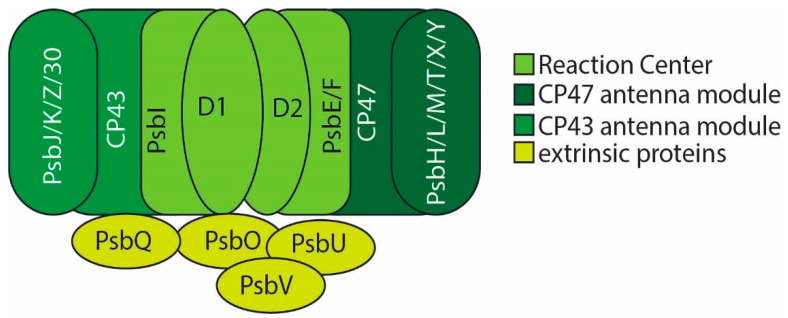
PSII monomer (PSII forms a dimer in vivo). Modules are colored as indicated.

Active PSII has a modular architecture, consisting of what can be described as four ‘modules’ (Figure 1). These include the membrane-intrinsic reaction center and chlorophyll-binding antennas, and the membrane-extrinsic thylakoid lumenal proteins. For a thorough discussion of the PSII structure-function relationship, see Müh et al. [18].

The PSII reaction center (RC) is localized on the D1 and D2 protein subunits. These subunits bind the P680 reaction center chlorophyll special pair as well as the primary electron transfer chain, including plastoquinones Q_A_ and Q_B_ and the catalytic Mn_4_CaO_5_ water-oxidizing complex (WOC or OEC for oxygen evolving center). The PsbI subunit and cytochrome b559 (made up of the PsbE and PsbF subunits) complete the reaction center. This reaction center complex has been isolated independently and is the smallest PSII sub-complex capable of charge separation [19,20,21]. It is notable, however, that the CP43 subunit also provides a ligand to the manganese cluster, and so, while capable of charge separation, the reaction center proteins alone are not sufficient to bind the catalytic metal center or perform water-oxidizing chemistry.

The two antenna modules bind chlorophyll to absorb light energy that is funneled into the reaction center. These modules consist of the CP47 and CP43 proteins and their associated low molecular weight subunits (which are also referred to as low-molecular mass or small membrane-intrinsic subunits). PsbH, PsbL, PsbM, PsbT, PsbX, and PsbY bind to CP47. CP43 is associated with the small transmembrane subunits PsbJ, PsbK, and PsbZ and Psb30 (Ycf12). These low-molecular weight subunits can be individually deleted without a loss of photosynthetic capacity, but stabilize and optimize PSII photochemistry, and some may play a regulatory role in the PSII lifecycle.

The last module consists of membrane-extrinsic proteins which bind PSII on the lumenal side of the thylakoid membrane and contribute to PSII stability and optimal performance. This module is the site of greatest variability between and among plants, algae, and cyanobacteria [22,23,24,25]. 

In addition to the active PSII subunits, there are assembly factors and accessory proteins involved in de novo biogenesis and repair following photodamage that are not part of the active PSII complex. Knowledge of these is almost certainly not complete, and they number as many if not more than the protein subunits of the active complex. These proteins, especially the Psb27 subunit, have been the focus of much recent research effort.

## 3. PSII Assembly

**Figure 2:**
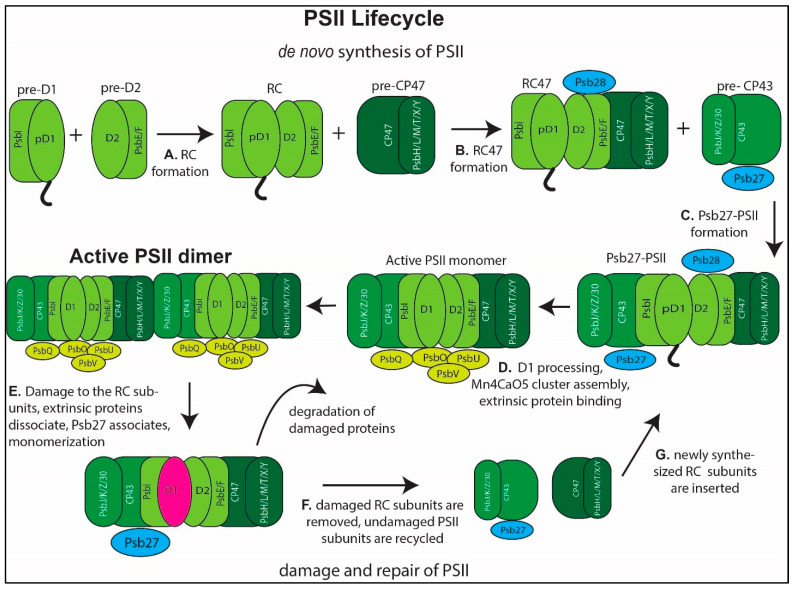
**PSII Lifecycle, simplified. Not all accessory assembly chaperones are illustrated**. (**A**–**D**) illustrate de novo synthesis, while (**D**–**G**) illustrate the repair cycle. (**A**) The pre-D1 and pre-D2 complexes come together to form the RC module. (**B**) RC and pre-CP47 modules come together to form the RC47 complex. Psb28 binds at this stage. (**C**) Psb27-PSII forms from pre-CP43 module and RC47. (**D**) The active PSII monomer is formed in a series of steps, which include processing of the D1 C-terminus, photo-assembly of the Mn_4_CaO_5_ cluster, and binding of extrinsic proteins PsbO, PsbV, PsbU, and PsbQ. The active PSII dimer forms following complete monomer assembly. (**E**) Following a photodamage event, the extrinsic proteins dissociate, Psb27 binds, and the dimer dissociates into monomers. (**F**) Damaged subunits are removed and proteolytically degraded. (**G**) Newly synthesized PSII subunits are inserted into recycled subunits to form a Psb27-PSII complex and the repair pathway re-joins the de novo synthesis pathway.

PSII assembly is thought to follow a highly ordered series of steps. The first step in PSII assembly is the formation of two pre-complexes, one containing pD1 and PsbI [26,27,28], and one containing D2 and cytochrome b559 [27,28,29,30,31] (Figure 2A, cytochrome b559 is composed of PsbE and PsbF). pD1 is a pre-processed form of D1. It has a 16 amino acid c-terminal extension that is cleaved by the protease **CtpA** (see Table 1 for details on bolded proteins) prior to assembly of the Mn_4_CaO_5_ cofactor [32,33]. The pre-D1 complex consists of the pD1 subunit and the PsbI subunit, as well as numerous translation and assembly chaperones that aid in ribosome binding to the *psbA* (gene coding for D1) transcript, insertion of the nascent peptide into the membrane, and chlorophyll binding to the newly inserted peptide. One such protein, **PratA**, is involved with the insertion of pD1 to the membrane and coordination of an initial Mn^2+^ ion to D1 [34,35,36]. It is also thought to be associated with specialized regions of the thylakoid membrane where PSII is synthesized and repaired [34,37]. The deletion of PratA was shown to be deficient in c-terminal processing of D1 [36], leading to the hypothesis that it facilitates access of the c-terminal protease, CtpA, to pD1. The rubredoxin **RubA** [21,31,38] also binds to D1 prior to its association with D2 and has been implicated in protection from photodamage to the reaction center during PSII assembly in the green alga *Chlamydomonas reinhardtii* [38]. 

Knoppova et al. found that Ycf39 and two HLIP family chlorophyll-binding proteins [39], **HliC** (ScpB) and **HliD** (ScpE), associate with D1 co-translationally [28]. The HLIP (high light-inducible) family of proteins is a family of chlorophyll-binding proteins related to the light-harvesting complex family of proteins in eukaryotes. In cyanobacteria, they are known to bind chlorophyll and stabilize the assembly of chlorophyll-binding proteins [39,40]. Ycf39, HliC and HliD form a complex which contains chlorophyll and β-carotene and is necessary for formation of the reaction center. This Ycf39-Hlip complex is also proposed to be involved in the delivery of recycled chlorophyll to newly synthesized D1 and protection from photodamage, as it interacts with the YidC/Alb3 membrane insertase complex as well as chlorophyll synthase [41,42]. **Pam68**, along with **Ycf48** and **Ycf39**, is implicated in co-translational insertion of chlorophyll to the reaction center proteins D1 and D2 [28,37,43,44]. Ycf48 is a soluble thylakoid membrane-associated protein associated with the D1 pre-complex and the reaction center complex [21,27,31,45,46,47,48,49], and it contains a conserved arginine patch that mediates its binding to integral membrane proteins [48]. These findings, in addition to the finding that apo-D1 does not accumulate [29], provide evidence that chlorophyll is co-translationally inserted into D1 as it is translocated into the membrane.

The reaction center forms (Figure 2A) [29,30,50,51] when the D1 and D2 pre-complexes bind together. **PsbP** (CyanoP), which binds to the D2 pre-complex [52], is thought to be involved in RC formation [22,53,54]. Recently, an RC complex was isolated from a strain of S6803 lacking CP47 that contains **PsbN** as well as the protein products of *slr0575* and *slr1470* [21]. This complex is on the de novo synthesis pathway, as active PSII does not form in this strain. However, a pre-D2 complex was also isolated from this strain which was associated with the protease FtsH2/FtsH3. This finding indicates that free-D2 levels are regulated by FtsH2/FtsH3.

The CP47 pre-complex contains PsbH, PsbL, PsbT, and possibly PsbM, PsbX, and PsbY [55,56]. The assembly factor **Psb35** also associates with the CP47 pre-complex and was shown to stabilize it and its association with **HliA** and **HliB** (ScpC and SpcD) [57]. Psb35 itself is a homolog of the cyanobacterial one helix domain/HLIP family of proteins, but it is unknown whether it binds chlorophyll. Pam68, in addition to its association with the pre-D1 complex, has been shown to be involved with co-translational insertion of chlorophyll into CP47 prior to PsbH binding [58].

In the next step of PSII assembly, the RC joins together with the CP47 pre-complex to form the RC47 complex (Figure 2B). RC47 is bound by HliA/HliB and **Psb28** [56,59,60,61,62,63,64]. While the deletion mutant of Psb28 is photoautotrophic, it is deficient in recovery from photodamage at high temperature [63] and in light/dark conditions [64], indicating a role in stabilizing PSII intermediate assembly and repair complexes. Recent structural studies are consistent with the role of Psb28 as stabilizing assembly intermediates and protecting them from photodamage as well (discussed below).

**Table 1:** PSII assembly factors. Psb subunits are listed alphabetically, followed by additional assembly factors, listed alphabetically.

Name	Function	S6803 Locus Tag	Homolog in *A. thaliana* or *C. reinhardtii*	Phenotype of Inactivation	Citations
**PsbN**	RC formation	smr0009	PsbN	Not significant in cyanobacteria	[21,49,65]
**PsbP (cyanoP)**	RC formation	sll1418	PsbP	Reduced O_2_ evolution, severe phenotype in low CaCl_2_	[22,52,54]
**Psb27**	Binds to CP43 during assembly	slr1645	Psb27	Defective photoactivation, sensitive to high light	[45,66,67,68,69,70,71,72,73,74]
**Psb28**	Binds to CP47/cytochrome b559 in RC complex and Psb27-PSII complex. Alters electron transfer properties to increase photoprotection	sll1398	Psb28	Susceptible to photoinhibition in high light	[46,56,59,60,63,64,71,73]
**Psb29**	Accumulation of FtsH2/FtsH3	sll1414	Psb29/Thf1	Impaired growth in high light, lower PSII efficiency	[75,76]
**Psb32**	?	sll1390	TLP18.3	Sensitive to photoinhibition	[77,78,79,80]
**Psb34**	Binds to RC47 and PSII-I prior to activation	ssl1498	?	N/A	[61,62,81]
**Psb35**	Pre-CP47	ssl2148	?	Lower CP47 accumulation, faster bleaching in dark	[57]
**CtpA**	C-terminal processing of D1	slr0008	CtpA	not photosynthetic, no Mn_4_CaO_5_ cluster formation	[32,66]
**HliA**	CP47 formation	ssl2542	?	Inhibited growth in high light	[39]
**HliB**	CP47 formation	ssr2595	?	Inhibited growth in high light	[39]
**HliC/ScpB**	Chlorophyll insertion	ssl1633	CAB/HLIP/ELIP family (counterpart of OHP1/OHP2)	Inhibited growth in high light, depleted chlorophyll	[21,28,39,40,82]
**HliD/ScpE**	Chlorophyll insertion	ssr1789	CAB/HLIP/ELIP family (counterpart to OHP1/OHP2)	Inhibited growth in high light, depleted chlorophyll	[21,28,39,40,41]
**Pam68**	Translation of CP47 and insertion of chlorophyll	sll0933	PAM68	Sensitive to high light, low temperature, fluctuating light	[43,44,58]
**PratA**	Mn^2+^ loading to D1 and D1 processing; thylakoid and plasma membrane connection	slr2048	LPA1	abnormal membranes, reduced PSII accumulation	[34,35,36,37]
**RubA**	D1/D2 assembly	slr2033	RBD1, *At1g54500*	Reduced PSII level and activity	[31,83]
**Slr0144-Slr0152**	PSII assembly associated	slr0144-slr0152	?	Slower growth and lower PSII activity	[47,84,85]
**Ycf39**	Pre-D2 stabilization, chlorophyll insertion	slr0399	HCF244	Decrease in thermotolerance	[21,28,41,44]
**Ycf48**	Insertion of D1, chlorophyll into D1, replacement of damaged D1, RC formation	slr2034	HCF136	Decrease in D1, PSII, increase in susceptibility to photoinhibition	[21,27,31,44,45,54,86]

RC47 binds to the CP43 pre-complex to form Psb27-PSII (Figure 2C). The CP43 pre-complex is composed of CP43, the **Psb27** assembly protein, PsbK, Psb30, and possibly PsbZ [55,70,72,87]. At this stage, the Psb27-PSII complex consists of most of the intrinsic membrane subunits of the PSII monomer. Sll0606 is involved in RC47 binding to CP43, and its deletion leads to a loss of photoautotrophy [88]. **Psb34** (Ssl1498), an HLIP-family protein with no pigment binding site, is also suggested to be involved in RC47 binding to CP43 [61,62,81], as it is found in both RC47 and Psb27-PSII. This subunit is suggested to be involved in later stages of PSII assembly following HLIP dissociation, as it seems to compete for binding sites on PSII intermediates, and in its deletion strain HliA/HliB increase [81]. It should be noted that, in [89], the subunit name Psb34 is assigned to a PSII subunit from *C. gracilis.* However, the sequence of that subunit was unable to be determined. Therefore, we defer to more recent publications that have assigned Psb34 to a PSII subunit with a known sequence and gene, Tsl0063 in *T. elongatus* or Ssl1498 in S6803, which is in a different position in the PSII structure from the unknown subunit.

In Figure 2D, the Psb27-PSII complex becomes the active PSII monomer. This step is presumably shared between the assembly and repair stages of the PSII lifecycle and requires several events to occur, the order of which are still unclear. These include the processing of the D1 C-terminus by CtpA, assembly of the Mn_4_CaO_5_ cofactor (photoactivation [90] discussed further below), dissociation of Psb28, dissociation of Psb27 [66,68,71], and assembly of the extrinsic proteins PsbO, PsbU, PsbV, and PsbQ (also called cyanoQ) [23,24,91]. 

It is also unclear at what stage some subunits found in the active PSII complex bind. In particular, PsbJ has not been found in intermediate complexes. PsbJ binds near the Q_B_ site and possibly does not bind until after the formation of Psb27-PSII, when it triggers Psb28 dissociation. The ΔPsbJ mutant retains Psb28 in the active PSII monomer and accumulates more of a Psb27-and Psb28-bound PSII complex [73,74]. This mutant also has a much longer lifetime of reduced Q_A_ as compared to the wild type. Therefore, it was proposed that PsbJ plays a role in forward electron flow from reduced Q_A_ to the plastoquinone pool [92,93]. This observation led to the hypothesis that binding of PsbJ is a regulatory step in PSII formation, only occurring following the release of Psb28.

In addition to the assembly factors described above, several genes have been implicated in PSII assembly and/or maintenance whose specific interactions with PSII have not been fully elucidated. The operon containing *slr0144-slr0152* [47,84,85] is implicated in optimal PSII function. Slr0151, in particular, is involved in D1 association with CP43 [84]. Its deletion causes lower PSII levels, impaired D1 replacement, and the reordering of the thylakoid membranes. **Psb32** is a transmembrane protein which was shown to minimize photodamage in cyanobacteria [78], but how it does so is unknown. **Psb29** was shown to contribute to optimal PSII maintenance in both S6803 and *Arabidopsis thaliana* [75] by interacting with the FtsH2/FtsH3 protease, which removes damaged PSII subunits.

Following assembly of the active PSII dimer, higher-level organization of antenna complexes and photosystems into supercomplexes takes place. In cyanobacteria, this involves phycobilisome docking and PSI-PSII-Phycobilisome supercomplex formation [64,94]. These inter-complex molecular interactions are likely to be important for optimal cellular metabolism and the highest photosynthetic activity.

## 4. PSII Repair

The repair cycle of Photosystem II is less well studied than PSII synthesis due to the inherently difficult nature of experimentally separating the two spatially and temporally overlapping processes. Deletion mutants of key photosynthetic proteins have been a crucial part of the elucidation of de novo PSII synthesis but are halted at certain points in the assembly of active PSII, and so never form active PSII to be damaged and subsequently repaired. What is well-established is that the lifetimes of PSII subunits are not all the same [40,95,96]. Experiments done by Vermaas and coworkers used the stable isotope ^15^N to determine the average lifetimes PSII subunits. They labeled cells with ^15^N for varying lengths of time to determine the rate of incorporation into various PSII subunits using bottom-up mass spectrometry. Subunits with a high rate of turnover had the highest rate of ^15^N incorporation. They found that the D1 subunit is turned over most frequently, with a half-life of less than an hour in cyanobacteria. This is followed by D2 with a half-life of about 3 h, CP43 at 6.5 h and CP47 around 11 h. Other proteins, such as Photosystem I subunits, are much more stable under normal conditions. Importantly, a similar labeling experiment was performed with ^15^N incorporation into chlorophyll, and it was found that it has a lifetime of 300 h in WT S6803 [96]. The high turnover rate of D1, and the stark contrast in lifetime between the PSII subunits, is of considerable interest, as it implicates that D1 is removed selectively from PSII and replaced, while other PSII proteins remain stably bound together. Additionally, chlorophyll is apparently recycled repeatedly among PSII and PSI complexes. Complementary experiments by Rögner and coworkers found that Psb27 is associated with both PSII assembly and repair intermediates using a similar strategy [67]. How the cell signals that certain subunits are damaged and selectively replaced, or if certain subunits are constitutively replaced at different rates, is not known. 

As for how the damaged protein subunits are selectively replaced, the assumption has been, due to the loose(r) association of the CP43 module and the existence of the RC47 complex, that the CP43 module dissociates, allowing for proteolytic degradation of D1 and re-synthesis and insertion at the RC47 level [51,97]. It is then assumed that, following re-insertion of D1 (and D2), that repair follows the same series of steps as de novo synthesis (Figure 2C,D). However, whether D1 insertion takes place at the dimer or monomer level has yet to be determined. A dimeric Psb27-PSII intermediate has been identified [69,74], and dimeric RC47 has been identified in a ΔCP43 mutant of S6803 [72], indicating that replacement of D1 may happen on the level of an RC47 dimer.

Recently, a complex that was a combination of the CP47 module and CP43 modules, called NRC (for No Reaction Center) [98], was isolated. Its abundance increased in the absence of protein synthesis and in high light, conditions that enhance PSII repair. This complex was hypothesized to be part of the repair cycle. Whether the CP47 and CP43 pre-complexes form a stable complex is disputed [99], but this is a topic that warrants further investigation.

## 5. Structural Advances in PSII Research

Cryo-electron microscopy (cryo-EM) has brought forth some of the most significant advances in our understanding of PSII assembly and function in recent years. An area of intense research focus and progress has been the elucidation of PSII structures from S6803 and also intermediate PSII complexes. Cryo-EM has provided an excellent means of studying such membrane protein complexes, as the technique does not require protein crystallization and allows for greater heterogeneity within samples. 

Protein crystals of PSII from thermophilic cyanobacteria have been extensively characterized by X-ray crystallography. However, the structure of PSII from the mesophilic S6803, on which many physiological and biophysical studies have been performed, had been elusive. Gisriel et al. [6] solved the cryo-EM structure of a dimeric, active PSII from S6803 to 1.93 Å. This structure is an important step in our understanding of PSII function, as many studies that model PSII based on biophysical data from S6803 assume that the structure and function is conserved between cyanobacterial species. However, as the authors note, there are significant differences in sequence between PSII from S6803 and the thermophilic organisms, and membrane proteins generally have different intermolecular interactions in mesophilic and thermophilic organisms. Importantly, this structure definitively shows the binding site of PsbQ, which was known to associate with active PSII in S6803 [77,91], but had not been captured in any cyanobacterial structure. PsbQ is found associated with the most highly active isolated PSII complexes [91]. However, the authors found that PsbQ binding does not affect the conformation of PSII when bound, hypothesizing that PsbQ stabilizes interactions of other membrane-extrinsic proteins with the PSII core, contributing to high activity in isolated complexes. This structure will certainly be of great value as a basis for computational studies to interpret the mechanism of PSII water-splitting, and the impact of point mutants in S6803.

Many intermediate PSII complexes have been isolated and characterized, but until recently, structural details of these complexes and their implications for PSII function were unknown. To better understand the role of Psb27 in the PSII lifecycle, Huang et al. [74] isolated a dimeric Psb27-PSII complex from *T. vulcanus* and determined the cryo-EM structure to 3.78 Å. The complex was isolated from a deletion mutant of the PsbV subunit, as increased assembly and repair intermediates are present in that strain. This dimeric Psb27-PSII does not have extrinsic proteins bound and it does not have an assembled Mn_4_CaO_5_ cluster. Psb27 binds to the E-loop of CP43, as evidenced previously by mass-spectrometry cross-linking data [70,72,87]. The authors found that there are limited molecular interactions between Psb27 and CP43, indicating relatively weak binding of the assembly factor. They demonstrate that Psb27 prevents binding of PsbO and PsbV by sterically hindering their association sites, which is consistent with data that report that Psb27 binds to PSII in Psb27-PSII to allow photoassembly of the Mn_4_CaO_5_ cluster to occur by preventing binding of extrinsic subunits [68]. Additionally, the authors did not find electron density corresponding to PsbY or PsbJ. PsbY has frequently been absent in PSII structures, which has been explained by the fact that it may easily dissociate due to its location on the periphery of PSII. PsbJ has been found to be absent in assembly intermediates that include Psb27. The authors suggest that structural perturbations from active PSII amongst subunits in Psb27-PSII may cause dissociation or prevent the association of PsbJ.

The dimeric Psb27-PSII in [74] may be the same species previously described by Grasse et al. [69] that was isolated from cyanobacteria grown at low temperature and high light. Because that dimeric Psb27-PSII was proposed to be part of the PSII repair cycle [69], and because the C-terminus of the D1 protein was unable to be modeled beyond Arg334 (the final residue in the mature peptide is Ala344) in the cryo-EM structure, the authors hypothesized that D1 in their sample may be partially proteolytically degraded as part of the first step of PSII repair. To test this hypothesis, they analyzed D1 c-terminal peptides from their sample through mass spectrometry and found that about 1% are proteolytically cleaved prior to the c-terminal Alanine 344 of the mature peptide. This finding confirms that the invisibility of the D1 C-terminus in their structure is due to its flexibility in the absence of a bound manganese cluster and extrinsic proteins, and not because it is degraded. However, the small amount of proteolytically cleaved D1 subunits does not preclude the possibility that this complex is part of the repair cycle, consisting of damaged PSII that has been partially disassembled. Interestingly, Zabret et al. [61] found that the D1 C-terminus of their monomeric Psb27-PSII structure (detailed below) can be modeled and is bound more closely to CP43 than in mature PSII. This difference may be due to a higher overall resolution of the structure, or to different stages of the complexes in the PSII lifecycle.

Zabret et al. [61] purified a PSII assembly intermediate containing Psb27, Psb28, and Psb34 from the ΔPsbJ mutant of *T. elongatus*, based on the observation from [73] that this mutant contains an increased amount of this intermediate sub-complex. They solved the cryo-EM structure of this complex, which they term PSII-I, to 2.9Å. In the structure, they found that the C-terminus of mature D1 is bound more closely to CP43 than in the active structure. They propose that this is to hold the D1 C-terminus in a conformation that favors photoassembly of the OEC. One cation was modeled at the OEC site, which could either be a Mn^2+^ or a Ca^2+^ cation. In addition to the difference in the D1 C-terminus described above, there is a slight discrepancy with the finding in the Huang Psb27-PSII structure in that they do not find a direct steric conflict with PsbO binding. PsbO has been found to be associated with Psb27-PSII in [70], so perhaps this also a difference between the dimeric, repair intermediate and the monomeric, Psb28-bound complex which may be the state of PSII just prior to photoactivation on the assembly pathway. On the acceptor side of PSII, they find that the D1 DE loop interaction with CP47 in active PSII is disturbed by Psb28 binding and that the CP47 C-terminus forms a β-sheet with Psb28. In addition, the Q_B_ binding site and the non-heme iron hydrogen bond network are altered from the active PSII structure, resulting in the absence of the bicarbonate ion and Q_B_. They found that these changes lead to changes in the PSII electron transfer pathway that reduce singlet O_2_ formation (discussed below). These findings suggest that Psb28 binds to PSII during its assembly to protect PSII from photodamage. Interestingly, Eaton-Rye found that PsbT interacts with the D1 DE loop in active PSII, and PsbT deletion causes increased photodamage [100,101,102], indicating a regulatory role of this loop in the repair cycle.

Xiao et al. also solved the structure of a Psb28 bound-PSII, as well as a Psb28-bound RC47 [62], to 3.14 Å resolution. They used a His-tagged Psb28 to isolate PSII intermediate complexes from both a ΔPsbV mutant and WT strain of *T. vulcanus*. Both the 28-PSII and the RC47 structure have Psb28, Psb34, and an unknown subunit bound that they were not able to model. This additional subunit was not found in the similar PSII-I structure in [61], but may be a second Psb34 copy. The authors found that Psb28 and Psb34 associate similarly to in the PSII-I structure, and consistent with the Zabret paper, there are changes in the quinone binding sites and H-bond network around the non-heme iron, with absence of the bicarbonate. They also observed a similar shift of the D1 C-terminus and increased flexibility. CP43, and the CP43 module as a whole is also shifted relative to its position in active PSII, consistent with [61,103], which the authors describe as a looser attachment. Additionally, alpha helices in D1, D2, and CP47 are changed to loops near the Psb28 binding on the cytoplasmic side. Overall, the PSII-I and Psb28-PSII structures are consistent with the binding position of Psb28 determined by Weisz et al. [59], although the precise attachment is slightly altered (see below). Intriguingly, the WT Psb28-PSII structure has Psb27 bound, but ΔPsbV does not. This finding led the authors to propose that Psb27 associates following Psb28 dissociation. However, it is not consistent with multiple findings that CP43 and Psb27 associate with one another prior to joining with RC47. PsbJ and PsbY were not found in either structure.

Xiao [62] and Zabret [61] both observed a distorted Q_B_ binding pocket and bicarbonate and heme iron binding site in the Psb28-bound PSII. These structures are consistent with the position of Psb28 that was identified by [59], and they find that, as proposed by Weisz et al., the donor side of PSII is perturbed by the presence of Psb28. Discrepancies between the crosslinking data and the structure could also have resulted from the fact that the location in the crosslinking paper was modeled off the active structure, while it was found that Psb28 binding causes significant structural changes in the acceptor (cytoplasmic) side of PSII. These structural alterations may slow electron transfer to the plastoquinone pool and change the midpoint redox potential of Q_A_/Q_A_^−•^, as proposed by Brinkert et al. [104], who showed that bicarbonate loss alters the *Em* of Q_A_/Q_A_^−•^ to a more positive value. This positive shift is predicted to increase the potential between P_680_^+•^/Q_A_^−•^ and P_680_^+•^/Pheo^−•^, which may favor direct recombination between P_680_^+•^/Q_A_^−•^ decrease back-reactions through P_680_^+•^/Pheo^−•^ recombination, reducing the probability of the formation of an RC triplet state [7,105,106,107]. Overall, this may lead to a decrease in the production of singlet O_2,_ consistent with the findings in Zabret et al. Additionally, photoactivation is also thought to decrease the potential of Q_A_/Q_A_^−•^ [108], so intermediate complexes such as the Psb28-bound PSII structures lacking OEC would also be protected by a similar mechanism. 

Higher levels of Psb27-PSII are also associated with higher NPQ [109]. This finding would be consistent with an overall model where Psb27 and Psb28 binding to PSII intermediates induces structural perturbations that protect the not-fully-assembled PSII from photodamage. An unanswered question in the role of Psb27 is that of Psb27 N-terminal lipidation and how it relates to the interaction between PSII and Psb27. Nowaczyk and coworkers initially suggested that Psb27 had an n-terminal lipid modification based on the presence of a lipobox motif next to the N-terminal cysteine of the mature peptide and confirmed a lipid attachment through mass spectrometry [67]. Recently, more focused mass spectrometry has provided a more detailed picture of the lipid attachment [110]. However, this lipid attachment was not resolved in the recent structures of Psb27-PSII, so how it mediates attachment of Psb27 to the thylakoid membrane and/or to PSII remains unclear.

## 6. Advances in Photoactivation

Assembly of the Mn_4_CaO_5_ cluster takes place through sequential binding and oxidation of Mn^2+^ ions, as well as the binding of Ca^2+^ and the deprotonation of water, using the same photochemical machinery for charge separation as active PSII. Photoactivation must take place during both de novo PSII assembly and repair, when the D1 subunit is replaced. This phenomenon has long been known and studied [12,90,108,111,112,113,114], but its exact mechanism is unclear. Recent PSII structures and studies have begun to describe this process in greater detail.

In one such structural study, Zhang et al. [115] solved the structure of apo-PSII (PSII without an intact Mn_4_CaO_5_ cluster) from *T. elongatus* using X-ray crystallography and found that it aligned closely to the active PSII structure, but without electron density corresponding to the OEC. The authors in this study intentionally removed the Mn_4_CaO_5_ cluster by treating crystals with hydroxylamine and EDTA, but the extrinsic PSII subunits were maintained. They propose that the lack of structural alteration of the OEC ligands in its absence means that the protein scaffold is already ‘pre-organized’ prior to Mn_4_CaO_5_ cluster assembly, and significant rearrangement does not need to take place for it to form. However, in another study, Gisriel et al. solved a structure of apo-PSII from S6803 [116] using cryo-EM and did find structural differences in the soluble domain of CP43 and the D1 C-terminus between their structure and that of active PSII. It is likely that this structure from S6803 lost the Mn_4_CaO_5_ cluster during sample preparation, unlike the Psb28-and Psb27-PSII structures described above, which are most likely assembly complexes. However, the study does provide insight into photoactivation, as the S6803 apo-PSII lacks the OEC and extrinsic PSII subunits and CP43 is shifted, as in [61,62,74]. These features suggest that the donor side of PSII does have some structural flexibility prior to photoactivation, and that rearrangement needs to take place during the assembly of the OEC. The S6803 apo-PSII structure is also lacking PsbY, PsbJ, and PsbZ, although whether they were lost in sample preparation is unknown. It is possible that the flexibility observed in the S6803 apo-PSII is not observed in the apo-PSII crystal structure due to the constraints of the crystals, as the OEC was removed after crystallization.

Computational modeling can complement structural studies and provide insight into structural perturbations and dynamics. Narzi et al. used a molecular dynamics simulation to find that structural rearrangements do need to occur after the first Mn^2+^ oxidation [117] for the manganese binding sites to form in apo-PSII. Their results are in agreement with the flexible nature of the D1 C-terminus found in [61,74,116], and they propose that alternate protonation of OEC-liganding residues contributes to flexibility in the D1 C-terminus as well. These results, together with Gisriel et al., also suggest that the structure solved by Zhang et al. was constrained by crystal contacts and does not represent the native structure of PSII prior to photoactivation. This study also agrees with a wealth of biophysical data that suggests a structural rearrangement of OEC ligands takes place during photoactivation [90,108,111,112,113,114,118].

The conditions necessary for optimal photoassembly, or photoactivation, of the manganese cluster have been the focus of recent work [118,119,120]. Photoactivation requires Ca^2+^ and is known to have a much lower quantum efficiency than photochemistry in active PSII leading to O_2_ evolution. Low quantum efficiency of photoactivation has been explained by the need for rearrangement of the ligand shell after the first Mn^2+^ oxidation, but also the need for acceptor side rearrangement to downshift Q_A_/Q_A_^−•^ potential [108]. Cl^-^ is necessary for photoactivation as well. Vinyard and coworkers found, by tracking photoactivation using EPR, that it tunes the *pK_a_* of residues around the OEC site, allowing for the necessary deprotonation [119]. In another recent study, Avramov et al. examined an over-expression strain of the Psb27 protein (OE27), as well as a deletion mutant of the PsbO protein for photoactivation. They found that, while both the ΔPsbO and OE27 strains had an increased optimal ratio of Ca^2+^/Mn^2+^, the quantum efficiency of photoactivation also increased in OE27, implicating a role for Psb27 beyond just blocking binding of extrinsic proteins as previously proposed [68]. The authors suggest Psb27 has a role in maintaining an optimal ligand configuration for photoassembly, in agreement with the altered CP43/D1 c-terminal conformation in [61,62]. How these results relate to the findings that Psb28- and Psb27- bound PSII have altered photochemistry, and when these subunits dissociate during the process, is yet to be determined.

## 7. Use of CRISPR and CRISPR Interference for Study of Photosynthetic Protein Complexes

CRISPR and its associated techniques have emerged as powerful tools to manipulate genes and gene expression in cyanobacteria [121,122,123,124,125]. Specifically in cyanobacteria, the nucleases Cas9 and Cas12a (Cpf1), and their DNase-dead counterparts have been used to great effect to efficiently make modifications. While these techniques are of great interest for metabolic engineering of cyanobacteria, they also have the potential to expedite and enable basic photosynthesis research. One advantage of CRISPR editing over more traditional genetic manipulation is that it is markerless, enabling successive mutations to be made without the need for multiple antibiotic resistance cassettes to be stacked or cells to be maintained on multiple antibiotics. Additionally, CRISPR editing allows for an expedited segregation process, which is an important consideration in an organism that has multiple genome copies.

Of recent interest in terms of Photosystem II research is CRISPR-interference, whereby a DNase-dead CRISPR-associated protein is targeted to a specific genomic locus (or loci) to block transcription. CRISPRi has emerged as a strategy to selectively inhibit photosynthetic genes. CRISPRi enables a particularly intriguing strategy for manipulation of photosynthetic proteins due to its reversibility and its potential to multiplex targets through incorporation of multiple guide RNAs [126,127]. In one example of this type of strategy, DNase-dead Cpf1 was introduced into *S. elongatus* UTEX 2973 as part of a CRISPRi system to target Photosystem I for inhibition [128]. Additionally, a plasmid-based CRISPRi system was introduced into S6803 [129] to inhibit the expression of the D1 protein of photosystem II, and Liu et al. [130] introduced a reversible CRISPRi system into S6803 by using a Rhamnose-theophylline inducible promoter system. This CRISPRi system was able to almost completely knock out PSII by targeting the D2 (*psbD* gene) protein, and it was fully reversible. These inhibition strategies introduce a new paradigm for the study of membrane protein complexes, in particular PSII, whereby its assembly can be reversibly halted at varying stages, depending on the gene targeted for inhibition.

## 8. Conclusions and Future Perspectives

A common point that has been further emphasized in recent studies is that subtle perturbations in PSII structure by either assembly factors or low-molecular-weight peripheral subunits have a significant impact on photochemistry. These perturbations can lead to overall efficiencies on a cellular level by protecting assembly intermediates from photodamage when necessary and optimizing photochemistry in active PSII. Along that theme, the impact of detergent solubilization on our current understanding of PSII function is not well described. A few alternate solubilization techniques that may preserve a more native membrane lipid arrangement, including lipid/protein nanodiscs [131,132,133] and SMALPs [134,135] have been explored, but it is an area that warrants more attention. 

A more complete structural and functional picture of the lifecycle of PSII is coming into focus, but there are most likely PSII assembly and repair factors that have not yet been identified. Open questions remain about several aspects of the lifecycle. These aspects include the mechanism of cofactor insertion into nascent PSII subunits and what signals that PSII is damaged and should be repaired [136,137]. Additionally, some PSII assembly factors have been identified whose function has yet to be fully determined. The study of these questions will lend insight into PSII functions and membrane protein complex assembly more broadly.

While knowledge about structures of isolated PSII intermediates has been expanded in recent studies, an open area of investigation is the cellular localization of PSII biogenesis and repair in cyanobacteria. The topic of localization of PSII biogenesis and repair is related to the question of the structure of the cyanobacterial membrane system. Liberton et al. [138] found through serial sectioning and electron tomography that the thylakoids form a completely independent membrane system from the plasma membrane. However, the thylakoid sheets do converge at certain cellular locations near the plasma membrane [139,140,141,142]. Whether these convergence zones are connected to the plasma membrane is debated, but they have been proposed to be the site of PSII biogenesis and/or repair [34]. An earlier study determined that reaction center proteins were localized to the plasma membrane of cyanobacteria, indicating that certain PSII proteins are inserted to the plasma membrane and then trafficked to the thylakoid to regulate PSII assembly, but that has since been disputed [143,144]. In plants, damaged PSII migrates to the thylakoid stroma lamellae from the grana stacks to be repaired [13], and the ‘thylakoid convergence zones’ are thought to be the location of an analogous process. Supporting this hypothesis is the fact that ribosomes are found at these sites, while phycobilisomes are not [142]. PratA, which binds to pD1, is found to be associated with an intermediate density membrane fraction between the plasma membrane and the thylakoid membrane [34,35,36,37]. The membrane fraction which contains PratA was proposed to be the site of the ‘thylakoid convergence zones’ (also called variously biogenesis centers, thylakoid centers, or thylapses [142]), as it also contained other PSII and chlorophyll biogenesis proteins. These studies were performed by separating membrane fractions by density, but an alternative approach was recently taken by Dahlgren et al. [145], which is a promising avenue for this type of investigation. The authors of that study utilized a proximity-based proteomics approach to identify components of different intracellular compartments. In this case, they used the APEX2 protein, a modified ascorbate peroxidase that catalyzes a reaction between biotin-phenol (BP) and hydrogen peroxide (H_2_O_2_) to create a BP radical that covalently attaches to proteins, fused to a known thylakoid lumen protein, PsbU, to selectively biotinylate proteins localized in the thylakoid lumen of *Synechococcus* sp. PCC 7002. Approaches such as this have great potential for answering questions of cellular localization and reinforcing findings by other methods.

Atomic force microscopy and hyperspectral confocal [146] studies of thylakoid fragments from *Thermosynechococcus elongatus*, *Synechococcus* sp. PCC 7002, and *Synechocystis* sp. PCC 6803 suggest that there are defined regions of the thylakoids that are differentiated by the arrangement of photosynthetic and respiratory complexes in cyanobacteria. This finding is corroborated by Casella et al. [147] in *Synechococcus elongatus* PCC 7942. The purpose of these differentiated membrane regions is yet to be determined, but the arrangement certainly has an impact on the diffusion of photosynthetic reactants and intermediates, such as quinones, as well as the spatial organization of PSII biogenesis and repair. However, it should also be noted that thylakoid arrangement varies drastically among cyanobacterial species [148], and so if and how these findings apply generally to other cyanobacteria is also unclear. Understanding the relative mobility of protein complexes and supercomplexes and assembly intermediates and their coordination will be key to understanding the purpose of these processes. Further advances in imaging, specifically electron microscopy correlated with light microscopy, and cryo-electron tomography or cryo-TEM on sections of cells, will shed light on this issue. One example of the potential of this kind of study is the in situ structure of a phycobilisome-PSII supercomplex from red algae [149]. New technologies, and new applications of technologies, will shed insight into our knowledge of PSII assembly, the process of membrane protein complex assembly more generally, and photosynthetic organisms on a systems level.
